# A cross-sectional study of the number and frequency of terms used to refer to knowledge translation in a body of health literature in 2006: a Tower of Babel?

**DOI:** 10.1186/1748-5908-5-16

**Published:** 2010-02-12

**Authors:** K Ann McKibbon, Cynthia Lokker, Nancy L Wilczynski, Donna Ciliska, Maureen Dobbins, David A Davis, R Brian Haynes, Sharon E Straus

**Affiliations:** 1 Health Information Research Unit, Department of Clinical Epidemiology and Biostatistics, McMaster University Faculty of Health Sciences, 1200 Main Street West, Hamilton, ON, Canada; 2 School of Nursing, McMaster University Faculty of Health Sciences, 1200 Main Street West, Hamilton, ON, Canada; 3 National Collaborating Centre for Methods and Tools, McMaster University, 1685 Main Street West, Hamilton, ON, Canada; 4 American Association of Medical Colleges, 2501 M Street Northwest, Washington DC, USA; 5 Li Ka Shing Knowledge Institute, St Michael's Hospital, University of Toronto, 30 Bond Street, Toronto, ON, Canada; 6 Family and Community Medicine, and Health Policy, Management and Evaluation, St Michael's Hospital, University of Toronto, 30 Bond Street, Toronto, ON, Canada

## Abstract

**Background**

The study of implementing research findings into practice is rapidly growing and has acquired many competing names (*e.g*., dissemination, uptake, utilization, translation) and contributing disciplines. The use of multiple terms across disciplines pose barriers to communication and progress for applying research findings. We sought to establish an inventory of terms describing this field and how often authors use them in a collection of health literature published in 2006.

**Methods**

We refer to this field as knowledge translation (KT). Terms describing aspects of KT and their definitions were collected from literature, the internet, reports, textbooks, and contact with experts. We compiled a database of KT and other articles by reading 12 healthcare journals representing multiple disciplines. All articles published in these journals in 2006 were categorized as being KT or not. The KT articles (all KT) were further categorized, if possible, for whether they described KT projects or implementations (KT application articles), or presented the theoretical basis, models, tools, methods, or techniques of KT (KT theory articles). Accuracy was checked using duplicate reading. Custom designed software determined how often KT terms were used in the titles and abstracts of articles categorized as being KT.

**Results**

A total of 2,603 articles were assessed, and 581 were identified as KT articles. Of these, 201 described KT applications, and 153 included KT theory. Of the 100 KT terms collected, 46 were used by the authors in the titles or abstracts of articles categorized as being KT. For all 581 KT articles, eight terms or term variations used by authors were highly discriminating for separating KT and non-KT articles (p < 0.001): implementation, adoption, quality improvement, dissemination, complex intervention (with multiple endings), implementation (within three words of) research, and complex intervention. More KT terms were associated with KT application articles (n = 13) and KT theory articles (n = 18).

**Conclusions**

We collected 100 terms describing KT research. Authors used 46 of them in titles and abstracts of KT articles. Of these, approximately half discriminated between KT and non-KT articles. Thus, the need for consolidation and consistent use of fewer terms related to KT research is evident.

## Background

Implementing important advances in health care knowledge and stopping invalidated or outmoded activities are vital to providing the best possible health care. Many people from a range of backgrounds and interests have begun to conduct research in this domain of implementing important knowledge in health care. The domain has many names; in this paper, we will refer to it as knowledge translation (KT), and base our use on the Canadian Institutes of Health Research (CIHR) definition:

'Knowledge translation is a dynamic and iterative process that includes synthesis, dissemination, exchange and ethically sound application of knowledge to improve the health of Canadians, provide more effective health services and products and strengthen the health care system.

'This process takes place within a complex system of interactions between researchers and knowledge users which may vary in intensity, complexity and level of engagement depending on the nature of the research and the findings as well as the needs of the particular knowledge user.' [1]

For the science and practice of KT to advance, ready access to KT literature across disciplines is essential. The KT literature is large and encompasses the spectrum of material from opinion pieces and editorials through controlled trials of interventions to improve clinical performance, formal modelling of the processes involved with KT, and qualitative studies of why and how interventions worked. This large body of literature includes two smaller subsets of material that have greater potential to inform KT practice and research. The larger of these two sets of literature includes the descriptions and data on KT implementations (*i.e*., application of specific research findings), and reports of projects and practices that have been implemented in clinical practice. Examples of this application literature are the kinds of studies that are included in the systematic review by Grimshaw and colleagues [2]. The second set of articles is the theoretical papers on models, tools, and methods to improve or implement KT. The number of articles on KT theory is smaller than the number of articles dealing with applications and implementations.

Although the term KT was used in adult education research in the 1950s, the term KT became used in the context of implementation of best evidence and was more recognized and studied as such in the late 1990s and early 2000s. In 2006, Graham and colleagues [3] recounted the varying concepts and terms related to the domain of KT, with special emphasis on how granting agencies describe and define KT. Davis [4] described the KT domain as interdisciplinary and thus provided some explanation for the variation in terminology that exists in KT research and application. In addition to being an emerging domain that crosses multiple disciplines, many countries are working in the KT area. They have developed terms unique to their countries and disciplines. Examples include Rogers' work [5] in diffusion of innovations in rural sociology starting in the 1940s in the United States, nursing research utilization also in the United States [6], KT and knowledge transfer in Canada [7], research capacity in the United Kingdom [8], and the know-do gap in Australia [9].

This plethora of terms and phrases for the processes of KT provides challenges for understanding and working with the KT literature and communication. Nowhere is this problem more evident than during report writing and information retrieval. The broad goal of the research described in this paper was to review and analyze KT terms and to develop information retrieval assistance for those searching for KT material. This retrieval assistance is to build search strategies, also called filters, that can be used to collect or sift the content of a major database such as Medline or Cumulative Index to Nursing and Allied Health Literature (CINAHL) so that all KT articles, and only KT articles, are retrieved. People interested in KT then can use the filtered literature and add their own content terms such as immunizations or smoking cessation. Search filters reduce the amount of information a searcher must go through while providing assurance that important material has not been missed.

An example of how filters can reduce the amount of non-relevant citations is the study of the use of computers for people with diabetes to help them in their disease management. Medline had 285 articles in April 2009 that were on this topic. Using methodologic filters for interventions [10], qualitative studies [11], and economics [12], brought the retrieval set down to 27 randomized controlled trials, 113 potential qualitative studies, or 12 economic analyses, respectively.

Search filters can be used to identify specific content and are available for such topics as mental health [13], palliative care [14], and nephrology [15]. Search filters, especially those designed to collect only specific research designs, are heavily used. Most filters are based on study methodology. The main goal of this funded project was to produce search filters for KT material; the results of which are reported in other papers. The current paper represents a sub-study and reports on KT term use in published reports--data that were collected as part of the filter production process.

In the production of the search filters, we sought to determine a list of KT terms and phrases used by authors, researchers, and practitioners. We also created a database of articles categorized as being about KT or not about KT. The database and list of KT terms were developed independently. The database and list were used in this study to determine which KT-related terms authors used in the titles and abstracts of articles and how often they are used. We also compared these rates of use of KT terms in the KT and non-KT articles in the database.

## Methods

Search filters can be developed using several methods [16]. The method used by our group involves establishing a database of articles, some of which are about KT and some of which are not about KT. The potential search filters are tested against this article database to determine how effective each is in retrieving those articles that are about KT and not retrieving those articles that are not about KT. This paper uses the filters data in a secondary analysis to determine how authors use terms related to KT in their articles. The following tasks were done to collect data for the production of the search filters:

1. Established working definitions of KT to develop an instruction manual for the research staff to use when categorizing articles as being about KT. The manual is included as a separate file to this paper.

2. Determined how many articles we would need in our analyses and projected how many journals we were going to read and the publication time frame for reading. At this step, we picked the journals to be read (journal list).

3. Collected terms and phrases used by practitioners and researchers that are related to KT (term list).

### Manual on categorization of articles

The reading criteria (see Additional File 1) were developed iteratively with input from all authors. We drew on the CIHR definition of KT as listed in the introduction section of this paper.

To operationalize this definition for categorizing the articles in the journals, we included the following notes that more fully define what aspects and specific content areas can be considered KT:

1. Administrative or educational interventions for policy and decision makers, practicing clinicians and patients/families/individuals, but not isolated groups of students, learning content to pass formal examinations/credentialing.

2. Application of evidence-based medicine/nursing/practice.

3. Process and outcomes assessments and appropriateness studies.

4. Quality of care studies where errors of omission or the gap between evidence and practice were addressed.

5. Quality improvement studies where errors of omission or the gap between evidence and practice were addressed.

6. Descriptions and methods of the processes in the production of systematic reviews, clinical practice guidelines, health technology assessments, and other knowledge syntheses.

7. Implementation of knowledge syntheses (systematic reviews and meta-analyses), clinical practice guidelines, or research findings.

8. Assessment of barriers and facilitating factors of innovations for groups of people/organizations.

9. Process and outcomes assessments when they involve a KT process and appropriateness studies.

10. Patient educational and clinician educational material.

Each article in the database was classified as being about KT or not about KT. An example of a KT paper is one by Shojana and colleagues [17]. They discuss their thoughts about the importance of supervision as a training method in clinical care. All papers that were categorized as being about KT were reviewed further to determine if they were about a KT application or about KT theory as defined below.

### KT application papers

Some of the KT articles were descriptions of application or implementation projects. Because this body of literature is especially important to practitioners and researchers we further categorized the papers that were identified as KT (as defined in the above step) as to whether each described a study or project in a specific setting or settings to assess some aspect of implementation of KT. These aspects can be barriers, factors that increase implementation, or a project to improve uptake of a specific intervention or knowledge area such as vaccinations, screening procedures, or smoking cessation approaches. A KT application paper can include projects that target individuals, institutions, or policy makers (*e.g*., a hospital, public health department, or country).

Rurup and colleagues [18] studied the factors that facilitated and hindered the institution of advance directives in three groups of people: the general population up to 60 years of age, the general population over 60 years of age, and the relatives of patients who died after euthanasia or assisted suicide. Their article is a KT article and also categorized as being a KT application paper. The paper by Shojana and colleagues [17] described above did not report a study or ground their thinking in a model of KT or education. Their paper was categorized as a KT article, but not a KT application of KT theory paper.

### KT theory papers

KT theory articles describe, use, or develop the general understanding of the KT process, tools, or theory. These articles, broadly speaking, give 'advice' or guidance to readers about how to understand or learn about KT or plan and 'do' KT projects. They can be considered to be the foundations of KT. They describe the theory, concepts, tools, or models of KT and delineate some of the issues and challenges of working in the area. The following topics are examples of the sort of paper that we tagged as KT theory: theories of KT; models or frameworks of KT or KT interventions; processes of KT; KT across disciplines (*e.g*., definitions); vocabulary of KT and scope; other theories that contribute to our understanding of KT, *e.g*., the theory of planned behaviour in a KT study [19].

These theory papers can include any component of KT: synthesis, diffusion, dissemination, implementation, uptake, awareness, agreement, adoption, or adherence. Lee [20] studied 15 nurses and their perceptions of the adoption process of personal digital devices in their daily work. Analysis used a framework based on Lewin's force field theory of change. This article is about KT, describes a KT application (handheald computer use in nursing care), and is based on a theory (Lewin's force field theory of change). Therefore it is placed into all three KT categories: all KT, KT application, and KT theory papers.

KAM and CL classified articles in duplicate, blinded to the other's decisions. Consensus was obtained via discussion between KAM and CL with input from the other authors as needed.

### Journals read

We chose to read journals for 2006 because it was the most recent full year of publication with complete indexing in Medline and CINAHL that was available at the start of our grants (early 2007). Sample size calculations based on data from Yao and colleagues [21] showed that a database with approximately 110 to 150 articles classified as being KT is needed to build and validate effective search filters. Initial analysis of the number of articles in individual journal titles gave us an estimate that 12 journals would provide sufficient numbers of KT articles.

Yao and colleagues[21] also show that to develop search filters articles need to come from journals that have relatively high and low numbers of articles that are in the categories for which filters are being built. To establish those journals that contain KT content, we used several strategies to ensure a broad coverage of titles. We produced a list of journals containing KT material from examining articles listed in the Atlantic Health Promotion Research Centre (AHPRC) KT database [22], the list of journals used in the production of a Health Services Research filter [12], and journals identified in the University of Alberta Research Utilization Resource Guide [23]). We also used work by Estabrooks and colleagues [22] who produced a bibliometric analysis describing the top 10 journals for knowledge utilization article publication from 1972 to 2001. We used their top 20 journals cited in the bibliographies. Using these resources we produced two lists of journals, one with journals likely to have considerable numbers of KT articles and the other with journals that include a relatively smaller proportion of KT articles. The journal lists were ordered by the number of expected KT articles.

Inclusion criteria for the journals chosen were that the journal had to be indexed in Medline and CINAHL and available online through the Health Sciences Library at McMaster University. We chose the top six journals for inclusion as the high-yield journals: *Annals of Internal Medicine, BMJ, Health Affairs, JAMA, Journal of Advanced Nursing*, and *Social Science and Medicine*. To sample titles from the lower-yielding journals, we used the list of journals generated using the above method and worked with those titles which had fewer than 20 KT articles for each title (132 journals). We divided the list into six segments with the same number of titles in each (22 journals) and by random number generation chose a title for reading. The journals with a lower proportion of KT articles were *Addiction, International Journal of Nursing Practice, Journal of Occupational and Environmental Medicine, Journal of the Medical Library Association*, *Nursing Inquiry*, and *Nursing Research*. Details on each title are in Table 1.

**Table 1:** Journals read and the number of articles categorized for all of 2006.

Journal Title	Journal Yield	Articles Read	All KT articles*	KT applications*	KT theory*
Addiction	Low	168	13 (8%)	9 (6%)	7 (4%)

Annals of Internal Medicine	High	268	116 (43%)	22 (8%)	7 (3%)

BMJ	High	520	150 (29%)	31 (6%)	23 (5%)

Health Affairs	High	205	40 (20%)	16 (8%)	12 (2%)

International Journal of Nursing Practice	Low	48	9 (19%)	5 (10%)	5 (10%)

JAMA	High	312	87 (29%)	18 (6%)	4 (1%)

Journal of Advanced Nursing	High	265	47 (18%)	35 (13%)	27 (10%)

Journal of Occupational and Environmental Medicine	Low	148	8 (5%)	4 (3%)	1 (1%)

Journal of the Medical Library Association	Low	59	16 (27%)	8(14%)	2 (3%)

Nursing Inquiry	Low	31	1 (3%)	0 (0%)	1 (3%)

Nursing Research	Low	49	7 (14%)	6 (12%)	7 (14%)

Social Science and Medicine	High	530	87 (16%)	47 (9%)	57 (11%)

Total of all articles read in the 12 journals		2603	581 (22%)	201 (8%)	153 (6%)

*Percent in each journal

Letters, editorials, comments and commentaries, book reviews, and news items were excluded. Journals are in alphabetical order.

We excluded letters, editorials, comments, news items, and book reviews from the 12 journals leaving a concentration of original and review articles. Each remaining article in the 2006 journal issues was considered for categorization as having KT content, and then further categorized, if warranted, as being a KT application or a KT theory paper.

### KT terms

We compiled lists of terms related to KT using published literature such as the work done by Graham and colleagues [3]; the internet; books and technical reports; and contact with authors, librarians, and content experts. Searching and compilation of the list of terms and collected definitions were done iteratively over six months with most of the collection done before development of the database of articles. Collection of terms and definitions continues and is independent of the work of categorization of the articles in the database. Terms are available on a public Wiki called WhatisKT [24].

### Analyses

Using custom-designed computer programs, we determined the number of articles containing each of the KT-related terms for all of the articles that we categorized as KT or non-KT. We chose to look at only the titles and abstracts of these articles for two reasons. First, titles and abstracts are the tools that authors provide and the indexers of bibliographic databases (such as Medline) use to enable information retrieval. Second, the title and abstract of a paper provide a summary of its content as a guide to whether the full text is worthwhile retrieving for further consideration. We determined if the use of the KT terms was more common in the titles and abstracts of KT articles than in the titles and abstracts of non-KT articles (chi-square test of proportions using Stata Intercooled 9.0 software). These data provided evidence of how often the terms related to KT were used by the authors producing KT papers and if this use was associated with being KT articles.

## Results

A total of 2,603 articles were reviewed in the 12 journals for all issues in 2006: 581 were tagged as having KT content, of which 201 were KT application articles, and 153 were KT theory articles (Table 1). On average, approximately one article in five (22% of all articles) was classified as KT; KT application articles (8% of all articles) and KT theory articles (6% of all articles) were less common. The journals performed as predicted based on projections of containing a larger or smaller number and proportion of articles that included KT content (Table 2). The high-yield journals averaged 25.8% of articles with KT material and the low-yield journals included a mean of 12.7% KT content.

**Table 2:** Journals read and the number of articles categorized for all of 2006 (Journals are in order of number of KT articles for both high- and low-yield journals).

Journals read	Articles Read	All KT articles*	KT applications*	KT theory*
**Journals with substantial numbers of KT articles**				

BMJ	520	150 (29%)	31 (6%)	23 (5%)

Annals of Internal Medicine	268	116 (43%)	22 (8%)	7 (3%)

JAMA	312	87 (29%)	18 (6%)	4 (1%)

Social Science and Medicine	530	87 (16%)	47 (9%)	57 (11%)

Journal of Advanced Nursing	265	47 (18%)	35 (13%)	27 (10%)

Health Affairs	205	40 (20%)	16 (8%)	12 (2%)

Mean percentage of KT articles in high yield journals	2,100	25.8%	8.3%	5.1%

**Journals with few KT articles**				

Journal of the Medical Library Association	59	16 (27%)	8(14%)	2 (3%)

Addiction	168	13 (8%)	9 (6%)	7 (4%)

International Journal of Nursing Practice	48	9 (19%)	5 (10%)	5 (10%)

Journal of Occupational and Environmental Medicine	148	8 (5%)	4 (3%)	1 (1%)

Nursing Research	49	7 (14%)	6 (12%)	7 (14%)

Nursing Inquiry	31	1 (3%)	0 (0%)	1 (3%)

Mean percentage of KT articles in low yield journals	503	12.7%	7.5%	5.8%

*Percent in each journal

Letters, editorials, comments and commentaries, book reviews, and news items were excluded.

We analyzed the 2,594 articles that were indexed in Medline (579 KT articles, 201 KT application articles, and 152 KT theory papers). The journals with the highest percentage of all KT content were *Annals of Internal Medicine *(43%), *JAMA *(29%) and *BMJ *(29%). The *Journal of the Medical Library Association *also had a high percentage of KT content (27%). *Journal of the Medical Library Association, Social Science and Medicine*, and *Nursing Research *had the highest percentage of KT application articles (14%, 13%, and 12% respectively). Nursing journals had the highest percentage of KT theory papers: *Nursing Research *(14%), *International Journal of Nursing Practice *(10%), and *Journal of Advanced Nursing *(10%). *Social Science and Medicine *also had a high proportion of KT theory articles (13%).

One hundred individual terms were identified as being equivalent or closely related to KT. Forty-six of these 100 KT terms (46%) were detected in the 2,954 articles indexed in Medline (*i.e*., all articles in the article database). Table 3 lists the 54 terms that were not included in the titles and abstracts. Of the 46 terms detected, only 19 terms were used 10 or more times in any of the articles (Table 4). Only 11 terms were used 10 or more times in the 579 KT articles.

**Table 3:** The 54 KT terms not found in the articles in the database (2,594 articles in 12 journals for all issues in 2006)

Applied dissemination	Know-do gap
Applied health research	Knowledge adoption
Audit and feedback	Knowledge brokering
Behavioural utilization	Knowledge communication
Cognitive application	Knowledge cycle
Comfirmatory utilization	Knowledge development and application
Disconfirmatory utilization	Knowledge exchange
Communicative utilization	Knowledge into use
Conceptual utilization	Knowledge mobile(z/s)ation
Cooperation or co-operation	Knowledge production and utilization
Effective dissemination	Knowledge to action
Effectiveness research	Knowledge transformation
Evaluation research	Knowledge translation
Evidence informed decision making	Knowledge uptake
Feedback and audit	Mindlines
Gap analysis	Mode 2 knowledge production
Guideline implementation	Populari(z/s)ation of research
Health care innovation or healthcare innovation	Potentially better practices
Impact	Product adoption and utilization
Implementation science	Research-practice gap
Information dissemination and utili(s/z)ation	Routini(z/s)ation
Innovation adoption and diffusion	Science communication
Innovation development process	Third mission
Innovation in health service delivery and organization	Translational medicine
Integrated knowledge translation	Translational science
Integrated knowledge transfer	Transmission of knowledge
Integrated knowledge Transformation	TRIP Turning research into practice

**Table 4:** KT terms and frequency of use by authors of KT articles.

		All KT articles		KT application articles		KT Theory articles	
		
Term tested	Total times used in database	Yes	No	Yes	No	Yes	no
		
		N = 579	N = 2,015	N = 210	N = 2,203	N = 152	N = 2,442
Use:	1,000	*31.0	40.7	*55.7	37.1	**49.3	37.9

Change:	359	15.7	13.3	*32.3	12.3	*27.0	13.0

Information	317	12.3	12.2	*22.4	11.4	***17.8	11.9

Change	197	**10.4	6.8	*22.4	6.4	*19.7	6.8

Policy	165	7.9	5.9	**11.9	5.9	*15.8	5.8

Evaluation	131	7.6	4.3	*15.9	4.2	13.2	4.5

Communication	84	2.8	3.4	5.5	3.0	4.6	3.2

Policies	67	3.6	2.3	**5.5	2.3	4.60	2.5

Implementation	63	*5.4	1.6	*11.4	1.7	*9.2	2.0

Utili(z/s)ation	52	***3.3	1.6	*5.5	1.7	*6.6	1.7

Adoption	31	*2.0	0.7	*6.0	0.8	4.6	1.0

Validation	19	0.3	0.8	0	0.8	1.3	0.7

Organi(z/s)ational innovation	19	**1.6	0.5	1.0	0.7	*3.3	0.6

Spread	18	0.3	0.8	1.0	0.7	1.3	0.7

Innovation	18	**1.6	0.4	***2.0	0.6	*3.9	0.5

Quality improvement	13	*1.2	0.1	*4.5	0.2	0	0.5

Dissemination	10	*1.2	0.1	*1.5	0.3	1.3	0.3

Diffusion	10	***0.9	0.2	1.0	0.3	*2.6	0.3

Best practice:	10	**1.0	0.2	**1.5	0.3	**2.0	0.3

Transfer	9	0.5	0.3	0.5	0.3	0.7	0.3

Patient safety	9	0.3	0.3	0.5	0.3	0	0.4

Best practice	8	0.7	0.2	**1.5	0.2	***1.3	0.2

Translation	7	0.2	0.3	0.5	0.3	0.7	0.2

Continuing education	7	0.5	0.2	1.5	0.2	0	0.3

Complex intervention:	6	*0.9	0.05	*2.0	0.09	*2.0	0.1

Total quality management	5	***0.2	0.1	0.5	0.2	**1.3	0.1

Sustainability	5	0.1	0.1	0.5	0.2	0	0.2

Implementation (w/3)research	4	*0.7	0	**1.0	0.087	*2.6	0

Complex intervention	4	*0.7	0	1.5	0.04	*2.0	0.04

Research utili(z/s)ation	3	0.5	0	0	0.1	*2.0	0

Institutionali(z/s)ation	3	**0.5	0	*1.0	0.04	0	0.1

Diffusion of innovation:	3	**0.5	0	0.5	0.087	*2.0	0

Action research	3	0.2	0.1	0.5	0.09	***0.7	0.08

Translational research	2	**0.3	0	0	0.087	**0.7	0.04

Quality assurance	2	2.2	0	0	0.087	**0.7	0.04

Participatory research	2	0	0.1	0	0.087	0	0.08

Participatory action research	2	0.2	0.05	***0.5	0.04	**0.7	0.04

Knowledge management	2	0.2	0.05	***0.5	0.04	0	0.08

Communities of Practice	2	0.2	0.05	***0.5	0.04	0	0.08

Capacity building	2	0	0.1	0	0.087	0	0.08

Technology transfer	1	0.2	0	0	0.04	0	0.04

Sociology of Knowledge	1	0	0.05	0	0.04	0	0.04

Service Innovation	1	0	0.05	**0.5	0	0	0.04

Research capacity	1	0	0.05	0	0.04	0	0.04

Policy Research	1	0	0.05	0	0.04	0	0.04

Opinion leader:	1	0.2	0	0	0.04	*0.7	0

Linkage and exchange	1	0.2	0	**0.5	0	0	0.04

Knowledge transfer	1	0.2	0	0	0.04	*0.7	0

Knowledge diffusion	1	0.2	0	0	0.04	*0.7	0

Knowledge (w/3)utili(z/s)ation	1	0.2	0	0	0.04	*0.7	0

Knowledge (w/3)synthesis	1	0	0.05	0	0.04	0	0.04

Knowledge (w/3)dissemination	1	0.2	0	0	0.04	0	0.04

Information Science	1	0.2	0	0	0.04	0	0.04

Effectiveness (w/3) research	1	0	0.05	0	0.04	0	0.04

All terms include various spelling and forms, *e.g*., utilization or utilisation and complex intervention or complex interventions. Data are provided in percentages of use of the term in the KT articles compared with the rest of the articles in the database. Statistical significance was tested with Chi-square analyses. Some terms are represented by more than one variation of the term, *e.g*., best practice and best practice: (truncated to get multiple endings).

* = highly significant p < 0.001

**moderately significant p = 0.001 to p < 0.01

***slightly significant p = 0.01 to p < 0.05

The proportion of how often a KT term was used in KT articles compared with non-KT articles was determined (Table 5). Chi-square analyses were used to determine if each term or term variation could be used to discriminate between KT and non-KT articles (Tables 5). For some terms, we used two or more variations of the term, *e.g*., complex intervention and complex intervention: truncated (allowing for both single and plural forms). From Table 5, for all KT articles, eight terms had high discrimination power (p < 0.001), seven terms had medium discrimination power (p > 0.001 to p = 0.01), and three terms had low discrimination power (p < 0.01 to p = 0.05). Of note, the term 'use:' truncated discriminated negatively--the use of the term was higher in the non-KT articles in the collection of all KT articles. Similarly for the KT application articles, 13 terms had high discrimination power, eight terms had medium discrimination power, and four terms had low discrimination power. For KT theory articles the discrimination numbers were 18 with high, six with medium, and three with low discrimination power.

**Table 5:** KT Terms that discriminate KT articles from non-KT articles for All KT, KT Applications, and KT Theory papers.

All KT articles (n = 579)	KT Application articles (n = 201)	KT Theory Articles (n = 152)
**High discrimination (p < 0.001)**		

Use: *(note negative association)* Implementation Adoption Quality improvement Dissemination Complex intervention: Implementation (w/3) research Complex intervention	Use: *(positive association)* Change: Information Change Evaluation Implementation Utiliz/sation Adoption Quality improvement Dissemination Complex intervention: Complex intervention Instititionaliz/sation*	Change: Change Policy Implementation Utiliz/sation Adoption Organiz/sational innovation Innovation Diffusion Complex intervention: Implementation (w/3) research Complex intervention Research utiliz/sation Diffusion of innovation Opinion leader* Knowledge transfer* Knowledge diffusion* Knowledge (w/3) utiliz/sation*
8 terms	13 terms	18 terms

**Medium discrimination (p > 0.001 to 0.01)**		

Change Organiz/sational innovation Innovation Best practice: Institutionali/sation Diffusion of innovation Translational research	Policy Policies Best practice: Best practice Continuing education Implementation (w/3) research Service innovation* Linkage and exchange*	Use: *(positive association)* Best practice: Total quality management* Translational research* Quality assurance* Participatory action research*
7 terms	8 terms	6 terms

**Low discrimination (p > 0.01 to <0.05)**		

Utiliz/sation Diffusion Total quality management*	Innovation Participatory action Research* Knowledge management* Communities of practice*	Information Best practice Action research*
3 terms	4 terms	3 terms

Terms are ordered by frequency in the full dataset (KT and non-KT articles) and some terms are included more than once with variations of spelling and term endings. P values are for the comparison of proportions of the term frequency in non-KT articles compared with the frequency of the term being in KT articles (Chi-square calculations).

z/s spelling indicate both English and US spellings

: indicates truncation

(w/3) means the second word appears within three words of the first word

*fewer than five instances of the term being used

## Discussion

Many journals contain substantial amounts of KT material, although the number and proportion varies by journal title and the features of the literature emphasized by the journal. Being able to readily and accurately identify the KT material is important. Information retrieval methods depend on a limited number of recognizable terms or phrases. In addition, understanding material is easier if a body of knowledge has standard terms and agreed upon definitions. We did not find consistent use of terms by authors in a large collection of articles concerned with KT. Less than half of the terms used to describe KT were present in KT articles. Only a relatively few terms were used in KT articles more often than in non-KT articles. Terms that discriminated between KT and non-KT articles, such as implementation, adoption, innovation, and complex intervention, were more common in the KT theory papers and to a lesser extent in the KT application articles than in all KT articles. Authors of KT theory papers are likely to be more versed in the intricacies of the domain of KT, and vocabulary issues will be more important to them.

KT is a relatively new discipline, and therefore hopefully in a state of flexibility in relation to its vocabulary and term use. Mitroff and Sagasti [25] in their 1973 paper on the use of the term 'stimulus' in the psychological literature summarized the challenges of a discipline's terms and concepts:

'In many respects, the most troublesome problems of any science centre around its most basic terms and fundamental concepts, and not around its more sophisticated concerns. Indeed to the extent that everything either follows from or is based on a discipline's basic terms and fundamental concepts, problems at a higher level can always be traced back to problems at a more fundamental level.'

With these thoughts in mind and recognition of the range of terms used in KT, we can choose to make changes to our work and how we report it to ensure that our research foundations are stronger and more readily accessible and understood. Building a standardized vocabulary for writing, collaborating, communicating, and information retrieval will facilitate assessing and applying our own evidence to our practices. If we take the time and effort to deal with the problems of our use of terms and definitions, we may be able to rectify our 'Tower of Babel' situation and streamline our communication across countries and disciplines. The challenge is not easy, and little experience exists on the 'right' number of terms and how to consolidate terms related to similar concepts.

Some of the steps that could make our literature more accessible and usable (and the domain of KT stronger and more effective) include the following:

1. We may need to seek assistance from colleagues in other disciplines: linguistics, terminology, information sciences, and philosophy are possible partners.

2. Setting standardized vocabularies and definitions for KT and its major concepts. This is a major undertaking, although other disciplines have done so. For example international clinicians, epidemiologists, and researchers met under the auspices of the World Health Organization to standardize definitions of drowning which are now used internationally [26].

3. Advocating a small set of terms to be used consistently by authors, educators, researchers, funders, and journal editors. Straus, Tetroe, and Graham [27] have started this process by defining what KT is and is not in a series of articles published in late 2009 in CMAJ.

4. Completing linguistic analyses of the literature and authors in KT, specifically lexical networks that show the relations between sets of synonymous or near-synonymous terms in a domain such as KT.

5. Determining if strategies such as search filters can be developed and tested that will facilitate information retrieval of KT literature.

6. Reviewing index procedures of large databases (*e.g*., Medline, Embase, CINAHL) to ensure that the literature of KT is readily available electronically. Currently, few indexing terms exist that reflect our work and thinking.

### Study limitations

The data in this study were obtained from a small collection of 12 journals that include KT material. Many other journals include KT content. For example, Estabrooks and colleagues [28] collected 603 articles on research utilization in nursing and found these articles came from 194 journals. We also searched for our term occurrences only in the title and abstract of the articles. Authors may have used other terms or term equivalents in the full text of the articles. Thirty-two terms or term variations were used five or fewer times making some of the statistical calculations unstable and we did not adjust for multiple statistical comparisons. These statistical issues would act to show more discrimination than is potentially present, and strengthen the case for more care with the use of KT terms.

Also of note, we used articles published in 2006, most of which were written in 2005 or early 2006. Terminology use may have changed since then. This paper was also completed without input from linguists, terminologists, or other language specialists. We did not assess co-occurrences of words and phrases. In addition, isolated words do not have the power to communicate as strongly or richly without their context and surrounding text.

## Summary

Authors writing about KT in 2006 used multiple terms to refer to their work making information retrieval and sharing of ideas and content difficult. Authors used only one-half of the terms identified in this study in their titles and abstracts of KT articles and of these, approximately only one-half of these terms discriminated between KT and non-KT articles. The category of all KT articles had the fewest number of terms that discriminated between KT and non-KT articles. KT application and KT theory categories had more terms that differentiated, that is, between KT application and non-KT application papers and also between KT theory and non-KT theory articles. The most consistent use of terms seemed to be in articles that dealt with the theoretical basis of KT and KT tools. But the need for consolidation and consistent use of fewer terms related to KT research is evident. The data in this article provide a starting point for further consideration of consensus building on standardizing terms and definitions and hopefully focussing on reducing the numbers. As a young and growing domain, we are in an ideal situation to do so.

## Competing interests

The authors declare that they have no competing interests.

## Authors' contributions

KAM, DAD, RBH, NLW, CL, and SES planned this project and provided input and guidance for the grant application. All authors guided implementation of the project with respect to the reading criteria for tagging the articles. DC, MD, RBH, SES, DAD and RBH provided training assistance in implementing the reading/categorizing guidelines and inter-rater reliability checks. KAM, CL, and NLW planned and carried out the analyses and interpretation of the data. All authors approved of the final content of the paper.

## Supplementary Material

Additional file 1 **Reading Criteria-Tower of Babel Study**. Reading Criteria--final version. Click here for file
