# Supplementary material for: A cross-sectional study of the number and frequency of terms used to refer to knowledge translation in a body of health literature in 2006: a Tower of Babel?
Source: Implement Sci. 2010 Feb 12;5:16. doi: 10.1186/1748-5908-5-16 (PMC2834600; doi:10.1186/1748-5908-5-16)
Supplement: Additional file 1 — Reading Criteria-Tower of Babel Study. Reading Criteria--final version. [file 1748-5908-5-16-S1.DOC]

**KT Hand Search Outline**

**Note: Reading must be done from the full text article—abstracts are not sufficient.**

**FORMAT and CONTENT-2 Sections/Decisions**

**Format Decisions**

**0.** Is this a letter, editorial, comment, erratum or news item (including invited PERSPECTIVES in Health Affairs)? If YES stop.

If NO continue to Question 1.

1. Is this article a **Knowledge Synthesis^1^**? If NO go to 2

(All knowledge synthesis are being tagged early. The content will be tagged in Q2.)

- 1. If YES (synthesis)—is it a **Review Article^2^**? If NO go to 1b.
     1. if YES (review) is it a **Systematic Review^3^**? Go to 1b.
  2. If YES (synthesis)—is it a **Clinical Practice Guideline^4^**? if NO go to 2

i. if YES (CPG) does it meet **CPG criteria^5^**? If NO go to 2

**Content Decisions**

2. Does this article have **knowledge translation^6^** (KT) content? If NO stop

- 1. if YES (KT)—is it a **KT APPLICATION^7^**? (KT “in action”)? If NO go to 2b
     - 1. if YES does it have data^8^? If NO go to 2b
          1. If YES (data) does it have outcome data^9^? If NO, 2b

If YES (outcome data) are the data…?

Qualitative? –continue

Quantitative? –Go to 2b

- 1. If YES (KT)—is it a **KT THEORY^10^** paper?

3. a) Is the unit of study clinicians (nurses, physicians, etc)

b) Is the unit of study patients or lay caregivers?

c) What is the unit of study if not the above: (free text choices include policy, populations, hospitals)

**Definitions for Scoring**

| **Term** | **Definition and Scoring Criteria** |
| --- | --- |
| **1. Knowledge Synthesis** | A review article or clinical practice guideline as indicated by a formal banner, a section heading, or indication in the title, abstract, methods section, introduction, or question with the intent to review, summarize or highlight, etc. the literature on a particular topic. |
| **2. Review** | Any full text article that is bannered ‘review, overview, or meta-analysis’ in the title or in a section heading, or it is indicated in the text of the article that the intention was to review, summarize, highlight, etc. the literature on a particular topic. Also classify as a review if data are combined from previously published studies. |
| **3. Systematic Review** | All 3 of the following criteria must be met for a review article to be classified as :   - The topic being reviewed must be clearly stated; - there must be a description of how the evidence on this topic was tracked down, from what sources; - Inclusion and exclusion criteria were applied to the articles included in the final paper   Only actual systematic reviews are included here. Articles ABOUT developing systematic reviews will be flagged in the “content” questions that follow. |
| **4. Clinical Practice Guideline (CPG)** | Definition: "…systematically developed statements to assist practitioner and patient decisions about appropriate health care for specific clinical circumstances.” (IOM) Also called standards, practice parameters, advisories, care paths, etc.  Only actual CPGs are included here. Articles ABOUT developing CPGs will be flagged in the “content” questions that follow. |
| **5. CPG Criteria** | For a CPG to “pass criteria”, the CPG must get at least one “YES” from both questions 1 AND 2.  1.Was there an explicit methods statement describing the process for developing the guidelines, (including   - a) methods of evidence assembly (indication of database searches), - b) method of review of studies (indication of a systematic process with criteria or expert panels) - c) one or more of: - organizations and individuals involved; - methods of formulating guidelines; and - methods of reaching agreement or consensus?   2. Was evidence cited in support of specific recommendations? (“Cited” means that a specific article was cited in relation to a recommendation, so that the evidence supporting the recommendation can be determined be checking the reference.) |
| **6. Knowledge Translation (KT)** | From CIHR:  “Knowledge translation is the exchange, synthesis and ethically-sound application of knowledge - within a complex system of interactions among researchers and users - to accelerate the capture of the benefits of research for persons through improved health, more effective services and products, and a strengthened health care system”  KT is synthesizing existing knowledge or implementing existing or unused knowledge (e.g., information, skill, techniques, process, tool, or technology) to those who are in a position to benefit from its application. The knowledge can be specific (eg implementing the best methods to increase smoking cessation for nurse practitioners or family physicians) or broad and general (increasing nursing hours to reduce hospital days and mortality).  KT must have more than basic measures of characteristics, there must be some form of action taken  KT includes the more specific areas of   - Educational interventions for practicing clinicians, patients/families/ individuals or policy/decision makers, administrators (but not isolated groups of students learning content to pass formal examinations/credentialing) - Application of Evidence-based medicine/practice - Quality of care studies - Quality improvement studies - Production of systematic reviews, CPGs, and other knowledge syntheses - Implementation of knowledge syntheses, CPGs or research findings - Assessment of barriers and facilitating factors of innovations or groups of people/organizations - Influence/impact of policy changes - Process and outcomes assessments and appropriateness studies   - **Appropriateness studies** (Studies (and reviews of studies) in which the objective is to determine whether individuals who are provided with a specific health care service (eg, carotid endarterectomy; beta-blockers) had the appropriate clinical indications for that service according to criteria.   - **Process Assessment (Health Care)** Studies or reviews of studies that assess the quality of health care by examining the process of care for people with a given health problem.   - **Outcome Assessment (Health Care)** Observational, longitudinal studies or reviews of such studies that assess the quality of health care in usual clinical practice settings by examining its effects on patient outcomes. These studies report data concerning the health status and events experienced by a group of patients who seek or are offered health care for a defined clinical condition (eg, diabetes) or set of clinical conditions or in a defined clinical service setting (eg, emergency room, primary care, community), comparing outcomes for patients who are treated according to the usual practice of clinicians making treatment choices under usual practice circumstances (ie, not under special research circumstances in which the investigator controls or directs who gets what). The objective of the study is to assess the consequences of various clinical decisions/management paths, not to assess the efficacy of a specific test or treatment. |
| **7. KT Application** | KT Application papers are papers that are identified as KT (in the above step) and **then describe a study or project in a specific setting or settings to assess some aspect of KT** such as barriers, factors that increase implementation, or a project to improve uptake of a specific intervention or knowledge area such as vaccinations, screening procedures, smoking cessation approaches, etc. A KT Application paper can include projects that target individuals or target institutions (eg a hospital, public health department, or country).  A general article that includes a brief example of an implementation in a specific setting (with or without data) to support general content is not to be tagged KT Application. |
| **8. Data** | Any kind of data on a completed or proposed project such as number of hours for a course, the number of times it was given or the number of attendees, the size of a hospital or organization, etc. |
| **9. Outcome Data** | Outcomes considered can be   - changes in knowledge, skills, attitudes and behaviours - improved or changed processes of care - improved or changed patient/system outcomes - changes in organizational culture, decision-making, work processes - changed policies and programs in institutions or communities - Improved capacity to use research in decision-making (individual and organizations) - Qualitative analysis producing themes etc are considered outcome data   The changes can be for individuals (e.g., a patient or health provider) or for a hospital or institution (e.g., more RN hours linked to fewer hospital days or deaths) |
| **10. KT Theory** | KT Theory articles describe, or develop the general understanding of the KT process or theory. These articles, broadly speaking, give “advice” or guidance to readers about how to understand or learn about KT or plan and “do” KT projects. They can be considered to be the foundations of KT. They describe the theory, concepts, or models of KT and delineate some of the issues and challenges of working in the area. Theoretical papers are useful for people who are starting to be active in the KT field or want more skills and knowledge of the area. The following topics are examples of the sort of paper that we are calling KT-Theory.   - Theories of KT - Models of KT - Processes of KT - Barriers to KT - Factors that facilitate KT - KT across disciplines (eg definitions) - Vocabulary of KT and scope - *Other theories that contribute to our understanding of KT*   These papers can include any “component” of KT: synthesis, diffusion, dissemination, implementation, uptake, awareness, agreement, adoption, and adherence.  **Read until the RESULTS section** of each paper or if no methods, check the whole paper. A KT Theory paper will have at least one of:   - Mention theory, science, model, or foundation in the abstract or title. For example this abstract ends with mentioning theory. (This paper would be both a KT Theory and KT Application paper):   “From this discussion emerge organizational requisites to conducting this work (e.g., development of key boundary-spanning figures; attention to the specific interests of potential linkage partners; translation efforts to demonstrate the value of participation; a continuous quality improvement approach featuring wide distribution of feedback in user-friendly form; flexibility, tact and patience), so that others can adapt and apply the linkage approach to manage HIV/AIDS or other problems. Finally, we explain how theory and practice have driven one another in this work.”  A title that would indicate a KT Theory paper would be:  “Clinical practice guidelines and the translation of knowledge: the science of continuing medical education”   - Include a banner or section heading that has the words theory, model, or foundation (or similar words). For example the Grol article (<http://www.mja.com.au/public/issues/180_06_150304/gro10753_fm.html>) has section headings “Theories and Models”, and “Models in…”. - At least 1 paragraph describing the concepts, theories, or models which relate to any element of the process of KT |
| **Unit of study** | Unit of study refers to the group targeted for an intervention in the case of clinicians and patients/lay caregivers. Other studies will assess policies or analyze populations, databases etc. Free text boxes must be filled with the following words:  Policy- defined as care that is delivered by making decisions that affect the healthcare system, the public health system, or the general health of populations. Policy has a 'trickle down' affect on individual patients and clinicians but the general decision making initially affects only systems, organizations, and populations.  Population-defined as looking at a group of people as a whole rather than individuals. Often based on databases.  Hospitals |

**Context for the CIHR definition as described in the CIHR Act: Knowledge translation is a broad concept. It encompasses all steps between the creation of new knowledge and its application to yield beneficial outcomes for society. This includes knowledge dissemination, communication, technology transfer, ethical context, knowledge management, knowledge utilization, two-way exchange between researchers and those who apply knowledge, implementation research, technology assessment, synthesis of results within a global context, development of consensus guidelines, and more.**
